# Performance assessment of novel chemiluminescence immunoassays for the detection of specific biomarkers for Alzheimer’s disease

**DOI:** 10.1002/alz.087324

**Published:** 2025-01-09

**Authors:** Philipp Arendt, Katharina Römpler, Britta Brix, Stephanie Franco, Viola Borchardt‐Lohölter, Anette Schulz, Mandy Busse, Stefan Busse

**Affiliations:** ^1^ Institute for Experimental Immunology affiliated to EUROIMMUN Medizinische Labordiagnostika AG, Lübeck, Schleswig‐Holstein Germany; ^2^ EUROIMMUN US Inc, Mountain Lakes, NJ USA; ^3^ Institute for Experimental Immunology, affiliated to EUROIMMUN Medizinische Labordiagnostika AG, Luebeck, Schleswig‐Holstein Germany; ^4^ Department for Experimental Obstetrics and Gynecology, University of Magdeburg, Magdeburg, Sachsen‐Anhalt Germany; ^5^ Department of Psychiatry, University of Magdeburg,, Magdeburg, Sachsen‐Anhalt Germany

## Abstract

**Background:**

To support diagnosis of Alzheimer’s disease (AD), concentrations of Aβ_1‐42_, tTau and pTau(181) and the Aβ_1‐42_/Aβ_1‐40_ ratio are determined in cerebrospinal fluid (CSF). To increase standardization and comparability between diagnostic laboratories, certified reference material (CRM) is used to evaluate the trueness of novel assays. Due to lack of CRMs for tTau, pTau(181), and Aβ_1‐40_, comparability may be assessed by comparing assays from different manufacturers. This study compares the performance of novel chemiluminescence immunoassays (ChLIAs) developed by EUROIMMUN with established chemiluminescence assays.

**Method:**

Biomarker concentrations were determined in 110 CSF samples (patients without known diagnosis; mean age 57.2 years, range 11–94 years; 49 female, 60 male, 1 unknown) using the Beta‐Amyloid (1‐42), Beta‐Amyloid (1‐40), Total‐Tau and pTau(181) ChLIAs (all EUROIMMUN) and the corresponding Lumipulse G assays (Fujirebio). The assays were performed according to the manufacturers’ instructions on the fully automated random‐access devices IDS‐i10 (Immunodiagnostic Systems) and LUMIPULSE G 600II analyzer (Fujirebio), respectively. Agreement of results obtained with the two chemiluminescence systems was calculated.

**Result:**

Overall agreement ranged from 89.0% to 97.3%. Agreement for the determination of normal biomarker concentrations was 93.4%–100% and 57.7%–94% for abnormal concentrations. The level of agreement was highest for Aβ_1‐42_ determination. Pearson’s regression coefficient revealed high correlation of results (R=0.82 to R=0.99 for Aβ_1‐42_ determination). While concentrations determined using EUROIMMUN assays were generally higher, numerical differences had minor influence on the diagnostic evaluation.

**Conclusion:**

Using the EUROIMMUN ChLIAs, AD‐specific biomarkers can be measured with high precision and stability on fully automated random‐access devices. The highest level of agreement and correlation was found for Aβ_1‐42_ determination as assays are aligned to the respective CRMs. This indicates the need to introduce CRMs for standardization of tTau, pTau(181), and Aβ_1‐40_ assays.

